# Mechanisms of telomere loss and their consequences for chromosome instability

**DOI:** 10.3389/fonc.2012.00135

**Published:** 2012-10-04

**Authors:** Keiko Muraki, Kristine Nyhan, Limei Han, John P. Murnane

**Affiliations:** Department of Radiation Oncology, University of California at San FranciscoSan Francisco, CA, USA

**Keywords:** telomere, gross chromosomal rearrangement, sister chromatid fusion, chromosome instability, double-strand break repair

## Abstract

The ends of chromosomes in mammals, called telomeres, are composed of a 6-bp repeat sequence, TTAGGG, which is added on by the enzyme telomerase. In combination with a protein complex called shelterin, these telomeric repeat sequences form a cap that protects the ends of chromosomes. Due to insufficient telomerase expression, telomeres shorten gradually with each cell division in human somatic cells, which limits the number of times they can divide. The extensive cell division involved in cancer cell progression therefore requires that cancer cells must acquire the ability to maintain telomeres, either through expression of telomerase, or through an alternative mechanism involving recombination. It is commonly thought that the source of many chromosome rearrangements in cancer cells is a result of the extensive telomere shortening that occurs prior to the expression of telomerase. However, despite the expression of telomerase, tumor cells can continue to show chromosome instability due to telomere loss. Dysfunctional telomeres in cancer cells can result from oncogene-induced replication stress, which results in double-strand breaks (DSBs) at fragile sites, including telomeres. DSBs near telomeres are especially prone to chromosome rearrangements, because telomeric regions are deficient in DSB repair. The deficiency in DSB repair near telomeres is also an important mechanism for ionizing radiation-induced replicative senescence in normal human cells. In addition, DSBs near telomeres can result in chromosome instability in mouse embryonic stem cells, suggesting that telomere loss can contribute to heritable chromosome rearrangements. Consistent with this possibility, telomeric regions in humans are highly heterogeneous, and chromosome rearrangements near telomeres are commonly involved in human genetic disease. Understanding the mechanisms of telomere loss will therefore provide important insights into both human cancer and genetic disease.

## TELOMERES

The genomes of eukaryotes are composed of linear DNA, which requires a mechanism to protect chromosome ends from being detected as DNA double-strand breaks (DSBs). The ends of linear chromosomes in eukaryotes therefore contain caps, called telomeres, that distinguish natural chromosome ends from DSBs (Blackburn et al., 2006). Telomeres prevent chromosome ends from activating DNA damage checkpoints and DSB repair pathways, and thereby prevent the degradation and fusion of chromosome ends. Mammalian telomeric DNA is composed of the TTAGGG repeat sequence with a short single-stranded 3′ overhang at its end. The single-stranded 3′ overhang is tucked back into a proximal complementary telomeric sequence to form the t-loop, which essentially hides and protects the free end (Griffith et al., 1999). A telomere-specific six-subunit protein complex called shelterin (TRF1, TRF2, RAP1, TIN2, TPP1, and POT1) plays a pivotal role in the protection of chromosome ends (Palm and de Lange, 2008), as well as facilitating telomere replication and the addition of telomeric repeat sequences. TRF1 and TRF2 bind specifically to double-stranded telomeric repeat sequences and recruit the other shelterin proteins and a variety of additional proteins to the telomeres. TRF2 protects chromosome ends by regulating the formation of the single-stranded 3′ overhang, while POT1, which binds specifically to the single-stranded 3′ overhang, facilitates end protection by promoting the formation of the t-loop (Hockemeyer et al., 2005). POT1 and its partner TPP1 are also important in the recruitment of telomerase to the telomere (Colgin et al., 2003; Xin et al., 2007). Telomerase is a reverse transcriptase that carries an RNA template that aligns with the end of the existing telomere to add additional telomeric repeat sequences (Blackburn et al., 2006). The regulation of access of telomerase to the telomere determines telomere length, which ranges from 2 to 20 kb on different human chromosomes (Lansdorp et al., 1996).

## THE MECHANISM OF TELOMERE SHORTENING DURING CELL DIVISION

Although telomeres are actively maintained in human germ line cells and embryonic stem cells through the expression of telomerase, telomeres shorten when human somatic cells divide due to insufficient telomerase expression (Harley et al., 1990; Hastie et al., 1990; Huffman et al., 2000). Approximately 50–200 bp of telomeric repeat sequences are lost each time a mammalian cell divides. This telomere shortening results from a combination of a failure to completely replicate the ends of linear DNA molecules, termed the “end replication problem,” and the processing of DNA that occurs on the ends of linear chromosomes.

During DNA replication, the end of the G-rich strand of the telomere is synthesized by leading strand synthesis, and the terminal of C-rich strand is replicated by lagging strand synthesis. Following DNA replication, the end of the leading strand will be blunt ended, and the 5′ end must therefore be resected to produce the single-stranded 3′ overhang (Lingner et al., 1995). However, the lagging strand is not completely replicated due to the presence of the RNA primer, which does not start at the very end of the chromosome (Olovnikov, 1973; Chow et al., 2012). As a result, telomere shortening occurs with each round of DNA replication.

Telomere shortening also results from the processing of ends of chromosomes following DNA replication, which is required to form functional telomeres. The single-stranded 3′ overhang is required for the association of the POT1/TPP1 complex and t-loop formation, and is therefore necessary for telomere end protection. In addition, the single-stranded 3′ overhang is used as a template for telomerase-mediated telomere elongation. Although the lagging daughter strands synthesized during DNA replication possess single-stranded 3′ overhangs at their ends soon after replication, the processing of the single-stranded 3′ overhang on the ends of leading strands is not completed until late S/G2 phase (Chow et al., 2012), consistent with the fact that telomeres are recognized as DNA damage in the G2 phase of the cell cycle (Verdun et al., 2005). The single-stranded 3′ overhang on the leading strand is generated by resection of the 5′ end of the leading strand by the Apollo exonuclease, which is recruited to telomeres through the interaction with TRF2 (Lam et al., 2010; Wu et al., 2010). The inhibition of Apollo results in a reduction in single-stranded 3′ overhangs, the appearance of DNA damage signals at telomeres, and chromatid fusions on the leading strand.

Like Apollo, EXO1 (Wu et al., 2010) and MRE11 (Larrivee et al., 2004; Chai et al., 2006; Deng et al., 2009) also possesses nuclease activity and contribute to the maintenance of the telomeric single-stranded 3′ overhang in a TRF2-dependent manner. Determining the exact role of MRE11 in maintaining single-stranded 3′ overhangs is complicated by the fact that MRE11 functions both as a double-stranded 3′ to 5′ exonuclease, and single-stranded endonuclease that acts on 5′ overhangs, 3′ flaps, 3′ branches, and closed hairpins (D’Amours and Jackson, 2002).

## THE ROLE OF DSB REPAIR IN TELOMERE MAINTENANCE AND CHROMOSOME FUSION

Understanding the mechanisms and consequences of the loss of telomere function requires knowledge of DSB repair pathways, both because DSB repair proteins are essential in telomere maintenance, and because dysfunctional telomeres are recognized as DSBs. There are three DSB repair pathways, homologous recombination repair (HRR; Moynahan and Jasin, 2010), classical non-homologous end joining (C-NHEJ, also referred to as canonical NHEJ; Lieber, 2010), and alternative NHEJ (A-NHEJ, also referred to as microhomology-mediated end joining, deletional NHEJ, or backup NHEJ; Nussenzweig and Nussenzweig, 2007; McVey and Lee, 2008; Zha et al., 2009; Mladenov and Iliakis, 2011). The initial steps in DSB recognition are similar for all three forms of DSB repair. The DSB is first recognized by the MRE11/RAD50/NBS1 (MRN) complex, leading to activation of ATM, which phosphorylates a large number of proteins involved in cell cycle regulation, chromatin remodeling, and DNA repair (Lavin, 2007).

The major DSB repair pathway in mammalian cells is C-NHEJ, which involves the direct joining of DNA ends using the proteins KU70, KU86, DNA-PKcs, XRCC4, LIG4, and XLF (Lieber, 2010). C-NHEJ can facilitate direct ligation with no processing of DSBs generated by I-*Sce*I endonuclease, although minimal processing can be required to clean up ends at DSBs generated by ionizing radiation or other means. HRR and A-NHEJ are actively suppressed in the initial phase of DSB repair by ATM (Rahal et al., 2008), which inhibits the resection of the DNA ends at the DSB by phosphorylating KU70 and KU86 (Fattah et al., 2010; Langerak et al., 2011; Sun et al., 2012), DNA-PKcs (Allen et al., 2003), 53BP1 (Cao et al., 2009; Bouwman et al., 2010; Bunting et al., 2010), the MRN complex (Langerak et al., 2011; Sun et al., 2012), and histone H2AX (Zha et al., 2010; Helmink et al., 2011). However, when DSBs are not repaired by C-NHEJ in a timely manner (Rass et al., 2009; Shibata et al., 2011), ATM then directs the processing of the ends of DSBs by MRE11 and CtIP to generate a single-stranded 3′ overhang (Sartori et al., 2007). These single-stranded 3′ overhangs are then used for repair by either the HRR or A-NHEJ pathways. For HRR, extensive resection is required by exonuclease EXO1 after processing by MRE11/CtIP (Steger et al., 2003; Sun et al., 2012; Tomimatsu et al., 2012). The long single-stranded 3′ overhang generated by resection is then used to pair with the homologous sequence in the sister chromatid, which then initiates replication through the region containing the DSB (Moynahan and Jasin, 2010). Error-free DSB repair with HRR is then completed by the resolution of the crossover junctions generated between the two sister chromatids. As a result of the requirement for the sister chromatid as a template, HRR can only be used for DSB repair in a limited portion of the cell cycle in which the region containing the DSB has already been replicated, but before chromosomes are condensed during mitosis. HRR involves a large number of proteins, including RAD51 and BRCA2. MRE11, CtIP, and BRCA1 are involved in generating the single-stranded 3′ overhang, while BRCA2 facilitates the binding of RAD51 to the single-stranded 3′ overhang, which mediates its pairing with its complementary sequence on the sister chromatid (Moynahan and Jasin, 2010).

Like HRR, A-NHEJ also requires the processing of DSBs to generate a single-stranded 3′ overhang for DSB repair. However, unlike HRR, this processing by MRE11 can be either ATM dependent or independent (Rass et al., 2009), and does not require further resection, as shown by the lack of a requirement for BRCA1 (Rass et al., 2009; Yun and Hiom, 2009). Moreover, rather than using the single-stranded 3′ overhang for pairing with the sister chromatid, A-NHEJ joins together sites within two single-stranded 3′ overhangs, which is often facilitated by microhomology (Guirouilh-Barbat et al., 2007; Yan et al., 2007; Bennardo et al., 2008; Rass et al., 2009; Xie et al., 2009). The requirement for the processing of DNA ends during A-NHEJ is demonstrated by the fact that A-NHEJ is commonly associated with deletions (Guirouilh-Barbat et al., 2004; Xie et al., 2009), and is the primary repair mechanism involved in chromosome rearrangements (Zhu et al., 2002; Guirouilh-Barbat et al., 2007; Weinstock et al., 2007; Zhang and Jasin, 2011). The pathway(s) involved in A-NHEJ are not well defined, although some of the proteins that have been reported to be involved in A-NHEJ are PARP, LIG3, XRCC1, MRE11, and CtIP ( Della-Maria et al., 2011; Simsek et al., 2011; Zhang and Jasin, 2011).

In addition to DNA repair, NHEJ proteins play essential roles in telomere function. KU70 protects chromosome ends from homologous recombination in mouse cells (Celli et al., 2006), while a deficiency in KU86 in human cells is lethal due to its role in suppressing HRR within telomeric repeat sequences (Wang et al., 2009). DNA-PKcs is also important in regulating the resection of the end of the leading strand following DNA replication, so that DNA-PKcs deficiency results in an increase in chromosome fusions that specifically involve the leading strand (Bailey et al., 2001). HRR proteins have also been found to be involved in telomere maintenance, including BRCA2, which facilitates RAD51 loading for telomere replication and cap formation (Badie et al., 2010).

## LOSS OF TELOMERE FUNCTION DUE TO A DEFICIENCY IN SHELTERIN PROTEINS

The inability to properly form caps on the ends of chromosomes has severe consequences for a cell even when telomeric repeat sequences are still present. A deficiency in TRF2 in mammalian cells results in extensive chromosome fusion and cell death due to the inability to maintain the single-stranded 3′ overhang required for t-loop formation (van Steensel et al., 1998). The chromosome fusions resulting from TRF2 deficiency are dependent on ATM (Karlseder et al., 2004), and occur through C-NHEJ, as shown by the requirement for LIG4 for fusion to occur (Smogorzewska et al., 2002). Chromosome fusions in TRF2-deficient cells are dependent upon the nuclease activity of MRE11, which mediates the degradation of the telomeric single-stranded 3′ overhang, demonstrating that TRF2 prevents chromosome fusions involving C-NHEJ by protecting the single-stranded 3′ overhang (Deng et al., 2009). The high frequency of chromosome fusions in TRF2-deficient cells has made this a popular approach for investigating the mechanisms of chromosome fusion in mammalian cells. However, although a deficiency in TRF2 provides valuable insights into telomere structure and function, it is not representative of the mechanism of chromosome fusion involved in all types of telomere dysfunction. Cells deficient in POT1/TPP1 also show an increase in chromosome fusions, although not to the same extent as with TRF2 deficiency (Rai et al., 2010). However, unlike with TRF2 deficiency, chromosome fusions in cells deficient in POT1/TPP1 are independent of the C-NHEJ proteins 53BP1 and LIG4, and therefore occur by A-NHEJ. Moreover, the chromosome fusions with POT1/TPP1 are dependent on ATR, not ATM (Denchi and de Lange, 2007; Guo et al., 2007; Deng et al., 2009). This difference between the mechanisms of chromosome fusion with deficiencies in TRF2 and POT1/TPP1 is consistent with the structure of the chromosome ends resulting from these deficiencies: the deficiency in TRF2 results in blunt ends, which are conducive for C-NHEJ, while the deficiency in POT1/TPP1 results in very long single-stranded 3′ overhangs (Hockemeyer et al., 2006; Wu et al., 2006), which are conducive for A-NHEJ.

Alternative NHEJ is also involved in chromosome fusions resulting from naturally occurring telomere shortening in mice. An initial study found that chromosome fusions occurring in mice that are deficient in the gene for the RNA template for telomerase, mTERC, required KU86 and DNA-PKcs (Espejel et al., 2002). However, subsequent studies with mTERC-deficient mice found that chromosome fusions occurred independently of LIG4, DNA-PKcs (Maser et al., 2007), and 53BP1 (Rai et al., 2010), leading to the conclusion that A-NHEJ is involved. Similarly, based on the prevalence of microhomology at the sites of chromosome fusions, A-NHEJ has been proposed as the primary mechanism responsible for chromosome fusions resulting from the loss of telomeric repeat sequences in human cells, either by gradual telomere shortening (Capper et al., 2007) or stochastic mechanisms (Lo et al., 2002b). The involvement of A-NHEJ suggests that chromosome fusions resulting from the loss of telomeric repeat sequences is commonly preceded by extensive resection, consistent with the large deletions demonstrated by DNA sequence analysis of the sites of chromosome fusion (Letsolo et al., 2010; Murnane, 2010).

## THE CONSEQUENCES OF EXCESSIVE TELOMERE SHORTENING IN NORMAL AND CANCER CELLS

The telomere shortening that occurs during cell division in human somatic cells can eventually result in replicative cell senescence or apoptosis if the telomeres become too short to protect the end of the chromosome (Sharpless and DePinho, 2007). Studies with human fibroblasts have shown that senescence occurs when the unprotected chromosome ends are recognized as DSBs (d’ Adda di Fagagna et al., 2003; Herbig et al., 2004; Sedelnikova et al., 2004; Zou et al., 2004), as shown by the presence of DSB repair complexes that co-localize with telomeres, called telomere dysfunction-induced foci (TIFs). The number of dysfunctional telomeres required for senescence may vary between different cell types. In fibroblasts, five different dysfunctional telomeres are required for senescence to occur (Kaul et al., 2012). The absence of chromosome fusions in senescent fibroblasts with dysfunctional telomeres demonstrated that these unprotected telomeres must be resistant to fusion, leading to the proposal that they must continue to have structures that prevent fusion despite being recognizable as DSBs (Kaul et al., 2012). However, the analysis of individual telomeres using single telomere end length analysis (STELA) demonstrated that chromosome fusions can occur at a low frequency in dividing human fibroblasts, and therefore that some fibroblasts containing fusions are capable of continued cell division (Capper et al., 2007). How these rare fibroblasts in the population adapt or fail to detect dysfunctional telomeres is not known.

The consequences of telomere shortening are dramatically different in cells with compromised cell cycle regulation. This was first demonstrated in human fibroblasts in which the p16 and p53 proteins in the senescence pathway were inactivated by viral proteins (Counter et al., 1992). The failure of these fibroblasts to senescence results in continued telomere shortening beyond the point that senescence would normally occur, eventually leading to “crisis,” which involves extensive chromosome fusion and cell death. However, rarely cells can survive crisis by acquiring the ability to maintain telomeres through the reactivation of telomerase (Counter et al., 1992) or through activation of an alternative pathway (ALT; Murnane et al., 1994; Bryan et al., 1995) involving recombination (Dunham et al., 2000), leading to the hypothesis that telomere maintenance is necessary for the extended cell division required for malignant cancer progression. Proof that telomerase expression is capable of providing cells with an indefinite life span was subsequently provided by the prolonged life span of human fibroblasts transfected with the gene for the catalytic subunit of telomerase, hTERT (Bodnar et al., 1998). A critical role for telomere maintenance in cancer is now well documented by the demonstration that nearly all malignant cancers have acquired the ability to maintain telomeres, in most cases through the expression of telomerase, although approximately 10% maintain telomeres through the ALT pathway (Shay and Wright, 2005). One apparent exception is chronic lymphocytic leukemia, in which telomere shortening continues to occur during disease progression (Lin et al., 2010).

Despite the fact that fibroblasts surviving crisis are capable of maintaining telomeres, they typically have highly rearranged chromosomes due to the extensive telomere shortening that occurred prior to expression of telomerase or ALT (Counter et al., 1992; Murnane et al., 1994). The structure of the chromosome fusions occurring in fibroblasts that fail to senesce has been extensively analyzed using STELA (Capper et al., 2007; Letsolo et al., 2010). The importance of the chromosome rearrangements caused by this extensive telomere shortening in cancer was demonstrated in mice that are deficient in the RNA subunit of telomerase, mTERC, and p53 (Artandi et al., 2000). Although mice with knockout of mTERC alone show an increase in cancer, the presence of mutations in p53, which allows for growth of cells with chromosome instability, accelerates carcinogenesis and shifts the spectrum toward carcinomas. Moreover, the chromosomes in these carcinomas demonstrated rearrangements consistent with chromosome instability resulting from telomere loss. These results demonstrated that telomere loss in telomerase-deficient cells lacking cell cycle regulation results in chromosome rearrangements leading to cancer. However, the cancers in these telomerase/p53-deficient mice were limited in that they did not occur in all tissues and did not fully develop into highly malignant tumors. The limited cancer phenotype in these mice results from the lack of telomerase activity, since Cre recombinase-mediated activation of telomerase activity results in a more complete tumor spectrum and bone metastases in these mice (Ding et al., 2012). These studies prove that both chromosome instability resulting from telomere loss, and the acquisition of the ability to actively maintain telomeres are important events that contribute to carcinogenesis.

**FIGURE 1:**
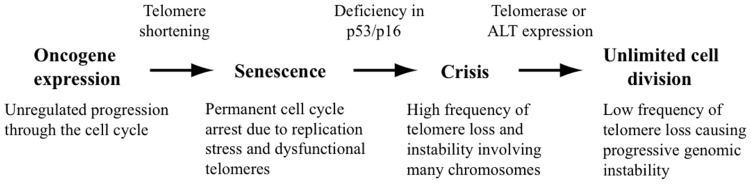
**Contribution of telomere loss to chromosome instability in cancer.** Oncogene expression causes unregulated cell division, resulting in replication stress and excessive telomere shortening. The very short telomeres or DSBs near telomeres that are caused by replication stress result in cell senescence. Mutations in the p53 and p16 proteins that are required for cell cycle regulation can allow cells to continue to divide, leading to cell crisis as a result of dysfunctional telomeres and extensive chromosome fusion. The activation of telomerase expression or the ALT pathway in rare cells allows for continued cell division, although the surviving cells will contain chromosome rearrangements as a result of telomere loss during crisis. Cells expressing telomerase will continue to experience a low rate of telomere loss due to a combination of replication stress causing DSBs near telomeres and a deficiency in DSB repair in subtelomeric regions.

Based on the above observations, it is often assumed that chromosome rearrangements resulting from telomere loss in cancer cells is a result of the transient period of telomere loss occurring during crisis before cancer cells acquire the ability to maintain their telomeres (**Figure 1**). However, it is now clear that not all chromosome rearrangements resulting from telomere dysfunction in cancer cells originate during crisis. Some cancers may arise in which telomerase activation occurs prior to crisis. Moreover, cancer cells commonly continue to demonstrate telomere dysfunction despite the expression of telomerase (see below). Although not as frequent as the telomere loss occurring during crisis, this lower rate of telomere loss is not lethal to the cell, and can therefore result in extensive chromosome rearrangements that can continue to occur throughout the lifetime of the tumor.

## STOCHASTIC MECHANISMS OF TELOMERE LOSS

In addition to gradual telomere shortening during cell division, the loss of telomere function can also occur as a result of stochastic events in which large blocks of telomeric repeat sequences are lost in a single rapid deletion event. Studies in yeast have demonstrated that a variety of mechanisms can result in stochastic telomere loss (Lustig, 2003). Stochastic telomere loss was initially described in the first report on the ability of human cells to maintain telomeres through the ALT pathway, which led to the prediction that this pathway involves recombination (Murnane et al., 1994). The correlation between the frequency of stochastic changes in telomere length and the frequency of chromosome fusions in this cell line also led to the conclusion that stochastic telomere loss was a mechanism for chromosome instability. As mentioned above, chromosome fusions resulting from stochastic telomere loss have also been observed in primary human fibroblasts, demonstrating that some fibroblasts in the population can continue to divide despite the presence of chromosomes with dysfunctional telomeres (Capper et al., 2007). Stochastic events are also responsible for the high rate of spontaneous telomere loss and chromosome fusion that occur in many human cancer cells despite the expression of telomerase. We first reported a high rate of telomere instability leading to chromosome fusions in the SQ9G squamous cell carcinoma cell line (Sprung et al., 1999a) and the EJ-30 bladder cell carcinoma cell line (Fouladi et al., 2000). Subsequent studies have demonstrated that this high rate of spontaneous telomere loss and chromosome fusion is common to many tumor cell lines and early passage cancer cells (Gisselsson et al., 2001; Nakamura et al., 2009). Thus, despite the expression of telomerase, telomere loss can continue to contribute to chromosome instability in cancer cells.

Errors occurring during DNA synthesis are likely to be an important mechanism for stochastic telomere loss in cancer cells. Telomeric DNA is a poor substrate for semi-conservative DNA replication, because telomeric DNA consists of a guanine-rich sequence on one strand and a cytosine-rich sequence on the other strand. As a result, telomeric DNA can take on non-B form higher order structures, such as G-quadruplexes. G-quadruplex DNA is much more stable than the B form of double-stranded DNA, and can pose a problem for DNA replication (Lipps and Rhodes, 2009). As a result, the 3′ to 5′ helicase activity of WRN, BLM (Mohaghegh et al., 2001), or RTEL1 (Ding et al., 2004) is required for replication of G-quadruplex DNA *in vitro*. In mammalian cells, WRN interacts with TRF2 and localizes at telomeres (Opresko et al., 2002). Consistent with an essential role for WRN in telomere function, WRN-deficient cells show chromosome fusions on the lagging strand, a DNA damage response, a growth defect, genome instability, and premature senescence. The expression of TERT and telomere elongation rescues these defects in WRN-deficient cells (Crabbe et al., 2004).

In addition to G-quadruplex structures, the D-loop structure created during t-loop formation also prevents replication fork progression, and resolution of the D-loop is required for telomere replication. WRN (Opresko et al., 2004) and RTEL1 (Barber et al., 2008) are also capable of resolving the D-loop structure. In addition to its helicase activity, WRN also possess a 3′ to 5′ exonuclease activity, and both activities cooperate to release the single-stranded 3′ invading overhang from the D-loop to permit the replication of the t-loop structures at telomeres (Opresko et al., 2004).

The replication of telomeric DNA is facilitated by the shelterin proteins TRF1 and TRF2, which bind to double-stranded telomeric DNA, and POT1, which binds to telomeric single-stranded 3′ overhangs. These proteins facilitate the replication of fork progression at telomeres by regulating the higher order structure of telomeric DNA. TRF1 plays an essential role in replication fork progression at telomeres, with the inhibition of TRF1 resulting in an ATR-mediated DNA damage response, chromosome breakage at telomeres, and chromosome instability (Sfeir et al., 2009). TRF2 induces positive supercoiling and modifies the topology of telomeric DNA (Amiard et al., 2007). This supercoiling-inducing activity of TRF2 is suggested to unwind DNA outside of TRF2 complexes. TRF2 relieves topological stress during telomere replication with the cooperation of Apollo and Top2-α (Ye et al., 2010). In yeast, Taz1, the ortholog of TRF1 and TRF2, is also required for replication of telomeric sequences (Miller et al., 2006). POT1 interacts with the telomeric single-stranded 3′overhang and prevents the formation of G-quadruplex structures (Zaug et al., 2005). POT1 also suppresses ATR activation at telomeres by blocking the binding of RPA to the single-stranded 3′ overhang (Denchi and de Lange, 2007). However, the suppression of ATR at telomeres may also prevent the detection of stalled replication forks, which could promote telomere loss and chromosome instability.

Stalled replication forks at telomeres can result from the presence of DNA damage in telomeric repeat sequences. This is an important mechanism for telomere loss, because telomeric regions are deficient in DNA repair. Ultraviolet light-induced pyrimidine dimers are poorly repaired at telomeres (Kruk et al., 1995). The guanine triplets in telomeric repeat sequences are also especially sensitive to oxidative modifications resulting from oxidative stress, and this oxidative damage at telomeres is also poorly repaired (Oikawa et al., 2001; Rhee et al., 2010). Moreover, oxidative damage causes the accumulation of single-strand breaks in G-rich strands, and these single-strand breaks are poorly repaired and persist longer at telomeres (Petersen et al., 1998). These oxidative base-modifications or single-strand breaks pose problems during DNA replication, as demonstrated by the telomere shortening and loss that occurs in cells undergoing oxidative stress (von Zglinicki, 2002).

The challenges of replicating telomeric regions can also result in the loss of telomeres under conditions of replication stress. Replication forks pause at regions with altered chromatin conformations in yeast, including telomeres, and require the RRM3 helicase to progress through these regions (Ivessa et al., 2002). A deficiency in RRM3 results in stalled replication forks in these regions, which subsequently leads to DSBs. Similarly, in mammalian cells, some DNA sequences also pose problems for DNA replication. The chromosome locations of these DNA sequences, known as fragile sites, form DSBs under conditions of replication stress (Debatisse et al., 2012). The regions near telomeres in mammalian cells have been demonstrated to be fragile sites in that chemically induced replication stress results in telomere instability (Sfeir et al., 2009). As a result, cellular alterations that affect the efficiency of the DNA replication machinery can promote stalled replication forks at telomeres and telomere loss.

## THE ROLE OF STOCHASTIC TELOMERE LOSS IN CHROMOSOME INSTABILITY IN CANCER

We investigated the role of stochastic telomere loss in chromosome instability in human cancer using clones of the telomerase-positive/p53-deficient EJ-30 human tumor cell line (Fouladi et al., 2000). These clones have the pNCT-tel plasmid containing the Herpes Simplex virus thymidine kinase (HSV-tk) gene integrated immediately adjacent to a telomere. Selection in ganciclovir for the loss of function of the HSV-tk gene serves as a marker for telomere loss on this chromosome. The rate of spontaneous loss of the HSV-tk gene near the telomere was found to be 10^-^^4^ events/cell/generation, which was more than 100-fold greater than at interstitial sites. In view of the fact that there are 96 telomeres, this means that one telomere is lost in this cell line approximately every 100 cell divisions. Unlike the overwhelming loss of telomeres occurring in crisis, the low frequency of telomere loss in cancer cells expressing telomerase is not lethal, although it provides a continued source of chromosome instability (**Figure 1**, see below).

We have proposed that this spontaneous telomere loss in human cancer cells results from stalled replication forks at telomeres due to oncogene-induced replication stress (Murnane, 2010). Oncogene expression has been demonstrated to result in replication stress (Halazonetis et al., 2008; Tsantoulis et al., 2008). The importance of replication stress in genomic instability in cancer is demonstrated by the prevalence of rearrangements at fragile sites in human cancer cells (Bignell et al., 2010). Although chromosome rearrangements can occur at any fragile site, the DSBs occurring near telomeres would be especially vulnerable to chromosome rearrangements due to a deficiency in NHEJ near telomeres (Miller et al., 2011; Fumagalli et al., 2012; Hewitt et al., 2012). Consistent with our model for oncogene-induced replication stress as a mechanism for telomere loss, oncogene expression has subsequently been shown to result in telomere dysfunction-induced senescence (TDIS) in normal human fibroblasts in culture, and *in vivo* in preneoplastic cells (Suram et al., 2012). The fact that the dysfunctional telomeres in the cells with TDIS still contained telomeric repeat sequences led the authors to conclude that irreparable DSBs near telomeres rather than telomere loss were likely to be responsible. In contrast, TDIS was not observed in malignant tumors. Therefore, although oncogene-induced replication stress would result in telomere loss in tumor cells, the lack of cell cycle checkpoints would allow tumor cells with chromosomes without telomeres to continue to grow, leading to chromosome instability.

## MECHANISMS OF CHROMOSOME INSTABILITY RESULTING FROM TELOMERE LOSS

Barbara McClintock first proposed over seventy years ago that telomere loss can result in chromosome fusion and instability as a result of breakage-fusion-bridge (B/F/B) cycles (McClintock, 1941). B/F/B cycles occur when chromosome fusions resulting from telomere loss produce chromosomes containing two centromeres. B/F/B cycles can occur either by fusion between two different chromosomes, or fusion between sister chromatids following the replication of the chromosome without a telomere (Murnane, 2006). The chromosome can form a bridge during anaphase when the two centromeres are pulled in opposite directions, causing the chromosome to break as the cell divides. With sister chromatid fusion, the chromosome breaks result in inverted repeats on the end of the chromosome in one daughter cell and a terminal deletion on the chromosome in the other daughter cell. Because the broken chromosomes still do not have a telomere on one end, following replication the sister chromatids can again fuse and break in the next cell cycle. This cycle continues until the chromosome is lost, the cell dies, or the chromosome acquires a new telomere, which usually occurs by translocation of the end of another chromosome (Artandi et al., 2000; Sabatier et al., 2005).

We have investigated the series of events involved in chromosome instability resulting from spontaneous telomere loss in clone B3 of the EJ-30 human tumor cell line in which the pNCT-tel plasmid is located immediately adjacent to a telomere on chromosome 16 (Fouladi et al., 2000; Lo et al., 2002a; Sabatier et al., 2005). Unlike other studies, this approach allows us to select for the loss of a single telomere, and thereby follow the series of events involved in chromosome instability, rather than attempting to reconstruct the consequences of telomere loss based on rearrangements involving random chromosomes. The results confirm that spontaneous telomere loss results in chromosome instability involving B/F/B cycles, and that these B/F/B cycles continue for many cell generations. DNA sequence analysis of the recombination site in several ganciclovir-resistant subclones demonstrated the presence of inverted repeats, indicating that telomere loss resulted in sister chromatid fusion. Evidence for B/F/B cycles involving sister chromatid fusions was also provided by the absence of a telomere on the marker chromosome 16 in many cells in the population after many cell generations (Fouladi et al., 2000), as well as by amplification of subtelomeric DNA (the region adjacent to the telomere) and anaphase bridges involving the marker chromosome 16 (Lo et al., 2002a).

The effect of B/F/B cycles on chromosomal instability in the EJ-30 clone B3 was further investigated by analyzing the mechanisms through which the marker chromosome 16 acquired a new telomere (Sabatier et al., 2005). Cytogenetic analysis revealed that after more than 20 cell generations, the marker chromosome 16 had acquired a new telomere in over 80% of the cells in the population, and that over half of the new telomeres were acquired through translocation of portions of other chromosomes. Almost a quarter of all telomere acquisition occurred through non-reciprocal translocations. These non-reciprocal translocations often resulted in loss of a telomere from the donor chromosome, thereby transferring the instability to the donor chromosome, which could then go on to acquire a new telomere from a third chromosome. In other cells in the population, the marker chromosome 16 had acquired a new telomere by translocations involving duplications of the arms of other chromosomes. While this does not result in chromosomal instability being passed on to other chromosomes, it does lead to allelic imbalance, which is associated with cancer progression

The above studies clearly demonstrate that B/F/B cycles resulting from telomere loss can generate many of the types of chromosome rearrangements commonly associated with human cancer. Evidence that B/F/B cycles are involved in human cancer is shown by the prominence of inverted repeats within amplified regions in human cancers (Ford and Fried, 1986; Guenthoer et al., 2011), which have been proposed to result from B/F/B cycles (Guenthoer et al., 2011). Cancer genome sequencing has also shown that inverted repeats are common in pancreatic cancer, leading to the proposal that telomere loss and B/F/B cycles play an important role in this disease (Campbell et al., 2010). Although amplified regions in cancer cells are not always located at their original locations, this does not exclude B/F/B cycles as a mechanism. Amplified regions containing inverted repeats are highly unstable and can form double-minute chromosomes (Singer et al., 2000) that can reintegrate at other locations (Windle et al., 1991; Coquelle et al., 1998).

## THE SENSITIVITY OF TELOMERIC REGIONS TO DSBs

**FIGURE 2:**
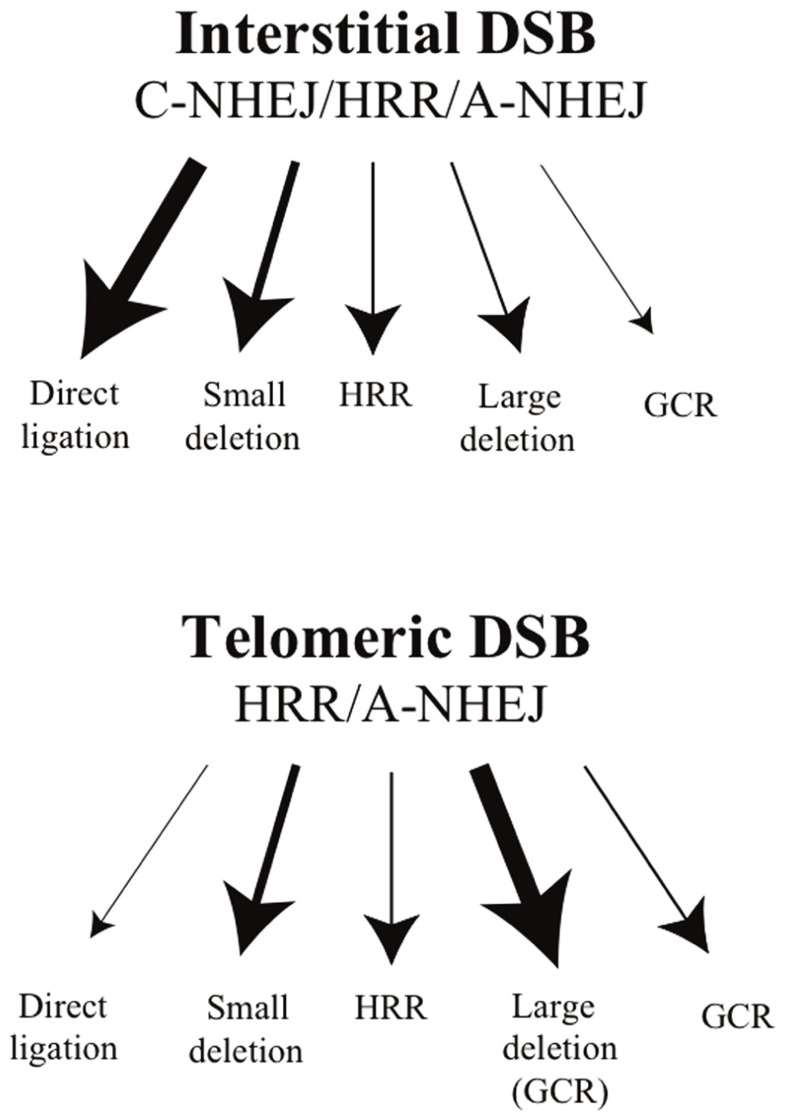
**The types of events resulting from interstitial and telomeric I-*Sce*I-induced DSBs.** DSB repair at interstitial sites occurs primarily by C-NHEJ, although some DSBs are also repaired by HRR, involving error-free repair, and A-NHEJ, which is associated with large deletions and GCRs. Direct ligation of the ends of DSBs without the loss of the I-*Sce*I site by C-NHEJ is the most common event at interstitial DSBs. Small deletions of a few base pairs that eliminate the I-*Sce*I site are the next most common event, followed by HRR, large deletions, and GCRs. The deficiency in NHEJ near telomeres is proposed to be due to inhibition of C-NHEJ, while the efficiency of HRR is unchanged. As a result, most DSBs near telomeres are repaired by A-NHEJ. Consistent with a predominant role for A-NHEJ in repair of telomeric DSBs, large deletions are the most common event at I-*Sce*I-induced DSBs near telomeres. Most of these large deletions would also result in GCRs, because they cause the loss of the telomere. GCRs that occur without large deletions are also greatly increased at DSBs near telomeres, while small deletions of a few base pairs occur at the same frequency as at interstitial DSBs.

To investigate the mechanisms of telomere loss in human cancer cells, we have analyzed the types of rearrangements resulting from DSBs near telomeres in clones of the EJ-30 human tumor cell line that contain the pNCT-tel or pNTIL-tel plasmids with an I-*Sce*I site that are integrated at either interstitial or telomeric sites (Zschenker et al., 2009). Consistent with earlier studies, we found that small deletions are the most common mutation when DSBs are induced at interstitial sites, while large deletions and gross chromosomal rearrangements (GCRs) are rarely observed (**Figure 2**). A similar frequency of small deletions was observed when DSBs are generated near telomeres. However, unlike interstitial DSBs, large deletions and GCRs are the most common type of DNA rearrangements at DSBs near telomeres (**Figure 2**). These GCRs included inverted repeats, translocations, and dicentric chromosomes, all of which can lead to further chromosome rearrangements or loss. These observations led us to be the first to conclude that telomeric regions are highly sensitive to DSBs. In a subsequent study, we demonstrated that the sensitivity of subtelomeric regions to DSBs extends at least 100 kb from the telomere (Kulkarni et al., 2010), and therefore subtelomeric regions represent a relatively large target for DSB-induced chromosomal instability (Kulkarni et al., 2010). An increased sensitivity to DSBs in telomeric regions has also been observed in studies in yeast, and these DSBs were found to be more likely to result in GCRs than DSBs at interstitial sites (Ricchetti et al., 2003).

**FIGURE 3:**
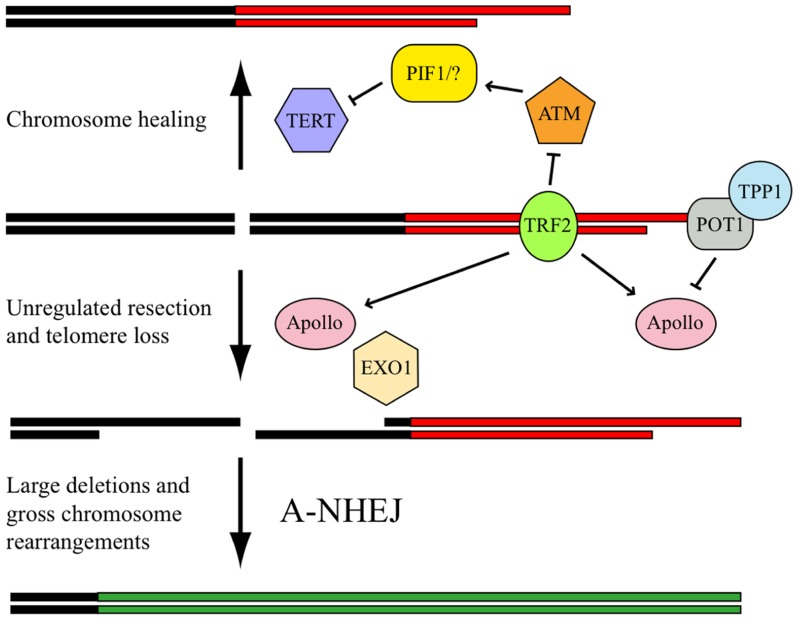
**Model for processing DSBs near telomeres.** The presence of TRF2 at the telomere (red horizontal lines) promotes chromosome healing by inhibiting ATM, which is required to activate PIF1 and/or other redundant proteins that prevent chromosome healing by binding to TERT, the catalytic subunit of telomerase. TRF2 also promotes the inappropriate processing of DSBs near telomeres by Apollo, which is then followed by extensive resection by EXO1. This function of Apollo is normally involved in the generation of single-stranded 3′ overhangs required for telomere formation. However, unlike telomeres (red horizontal lines), in which resection is limited by POT1/TPP1 binding to the single-stranded telomeric DNA, resection of DSBs in subtelomeric regions (black horizontal lines) continues unabated due to the inability of POT1/TPP1 to bind to non-telomeric DNA. The inappropriate processing of the DSB results in large deletions and/or GCRs with other chromosomes (green horizontal lines) through a mechanism involving A-NHEJ.

We next investigated the frequency of DSB repair at interstitial and telomeric sites using clones of the EJ-30 tumor cell line in which DSB repair is monitored by the activation of a gene for green fluorescent protein (Miller et al., 2011). No difference was observed in HRR for telomeric and interstitial DSBs; however, the frequency of NHEJ at telomeric DSBs was found to be much lower than that observed at interstitial DSBs. We have hypothesized (Miller et al., 2011) that this deficiency in NHEJ near telomeres is due to *cis*-acting telomere-binding proteins whose presence at telomeric regions causes DSBs to be processed as though they were telomeres (**Figure 3**). Following DNA replication, the leading strand at the end of the chromosome must be resected to generate a single-stranded 3′ overhang. This resection by either Apollo or MRE11 is regulated by TRF2 (Zhu et al., 2000; Deng et al., 2009; Lam et al., 2010; Wu et al., 2010), and is limited by the binding of POT1/TPP1 to the single-stranded 3′ overhang and formation of the t-loop. Our model proposes that TRF2 similarly directs the inappropriate resection of DSBs occurring near telomeres; however, because the single-stranded ends that are generated by resection are not composed of telomeric repeat sequences, POT1/TPP1 cannot bind to limit resection. As a result, excessive degradation occurs at the DSB. Although these large single-stranded regions at the DSB could facilitate HRR during G2 phase, they would inhibit C-NHEJ in other parts of the cell cycle, leading to telomere loss and GCRs involving A-NHEJ.

Consistent with our results in human tumor cells, two recent studies have reported that irreparable DSBs near telomeres in mammalian cells contribute to aging and stress-induced senescence (Fumagalli et al., 2012; Hewitt et al., 2012). Human cells exposed to ionizing radiation in culture and mouse cells *in vivo* demonstrate persistent DSB repair foci near telomeres, which correlate with stress-induced senescence. Similarly, aging normal tissues also accumulate DSB repair foci near telomeres. The ectopic localization of TRF2 caused a delay in repair of interstitial DSBs (Fumagalli et al., 2012), consistent with our model that TRF2 is responsible for the inhibition of repair of DSBs near telomeres. Moreover, this study demonstrated that the presence of telomeric repeat sequences inhibited the recruitment of the NHEJ protein LIG4 in yeast (Fumagalli et al., 2012), consistent with our results demonstrating a deficiency in NHEJ near telomeres in human tumor cells. Although not addressed in either study, the size of the target can be estimated from the frequency of persistent DSBs. The frequency of DSBs generated by X-rays is approximately 25–40 per Gy (Costes et al., 2010). These studies found approximately two to three persistent γH2AX foci after 20 Gy of X-rays, which represents 0.25–0.6% of the DSBs initially generated in these cells after 20 Gy. However, as was pointed out (Fumagalli et al., 2012), a target composed of telomeric repeat sequences represents only approximately 0.02% of the total human genomic DNA (12 kb average telomere length ×92 telomeres 6 × 10^9^ bp per genome = 0.018%). The target size that results in persistent γH2AX foci is therefore 14–33 times larger (168–396 kb per telomere) than the telomeric repeat sequences themselves, consistent with our model that subtelomeric sequences are deficient in DSB repair.

## CHROMOSOME HEALING IN RESPONSE TO DSBs NEAR TELOMERES

We have also investigated the effect of DSBs near telomeres on chromosome instability in mouse embryonic stem (ES) cells. As in our studies with the EJ-30 tumor cell line, these studies used I-*Sce*I-induced DSBs and plasmids containing the HSV-tk gene integrated immediately adjacent to a telomere (Sprung et al., 1999b; Lo et al., 2002b; Gao et al., 2008; Reynolds et al., 2011). The results were similar in many respects to those obtained with EJ-30, in that two of the most common rearrangements resulting from DSBs near telomeres are large deletions and sister chromatid fusions. In addition, the sister chromatid fusions initiated B/F/B cycles, which resulted in amplification of subtelomeric regions and translocations, although the B/F/B cycles in mouse ES cells were much shorter in duration than in human tumor cells. The presence of B/F/B cycles in mouse ES cells is important in that it demonstrated for the first time that chromosome instability resulting from telomere loss is not confined to tumor cells. An important difference we observed between mouse ES cells and the EJ-30 human tumor cell line is the prevalence of chromosome healing in mouse ES cells, which involves the addition of a new telomere at the site of the DSB. Chromosome healing accounted for only approximately 1% of the total rearrangements resulting from DSBs near telomeres in the EJ-30 clones, but accounted for approximately one-third of the rearrangements in mouse ES cells. Unlike chromosome fusions, which primarily occurred following extensive degradation at the site of the DSB, chromosome healing nearly always occurred at the site of the DSB, and therefore prevented the extensive degradation and chromosome instability resulting from DSBs near telomeres. Because chromosome healing has not been observed by us or others at I-*Sce*I- or ionizing radiation-induced DSBs at interstitial sites (Latre et al., 2004; Rebuzzini et al., 2005; Varga and Aplan, 2005; Honma et al., 2007), we proposed that chromosome healing in mouse ES cells serves as an important mechanism to compensate for the deficiency in DSB repair near telomeres. We also proposed that the apparent deficiency in chromosome healing in human tumor cells could contribute to the chromosome instability resulting from spontaneous telomere loss in human tumor cells.

Chromosome healing has been studied extensively in yeast, although very little is known about the regulation of chromosome healing in mammalian cells. In yeast, chromosome healing is inhibited by PIF1, and mutations in PIF1 result in a 1000-fold increase in chromosome healing (Schulz and Zakian, 1994). PIF1 mutations also result in telomere elongation, demonstrating that PIF1 regulates telomerase. Consistent with this conclusion, PIF1 binds to TERT, and mutations in TERT that influence PIF1 binding result in telomere elongation similar to cells deficient in PIF1 (Eugster et al., 2006). The inhibition of chromosome healing requires the phosphorylation of PIF1 by MEC1 in response to DSBs (Makovets and Blackburn, 2009). This inhibition of PIF1 in response to DSBs has been proposed as a mechanism for preventing PIF1 from interfering with DSB repair and causing terminal deletions (Zhou et al., 2000).

The high frequency of chromosome healing in mouse ES cells has allowed us to investigate the mechanism of chromosome healing in mammalian cells. As in yeast, we demonstrated that chromosome healing is performed by telomerase, although cells that have acquired the ability to maintain telomeres through the ALT pathway are also capable of performing chromosome healing (Gao et al., 2008). However, unlike yeast, the inhibition of PIF1 had no effect on chromosome healing (Reynolds et al., 2011), despite the fact that human PIF1 binds to TERT, as it does in yeast (Mateyak and Zakian, 2006; Snow et al., 2007). Based on these results, it has been proposed that the regulation of chromosome healing is likely to be redundant in mammalian cells (Snow et al., 2007; Reynolds et al., 2011). The prevalence of chromosome healing near telomeres could result from the inactivation of ATM by TRF2 (Karlseder et al., 2004), since the activation of PIF1 and other proteins for chromosome healing could require phosphorylation by ATM (**Figure 2**), similar to the activation by MEC1 in yeast. Although chromosome healing would result in terminal deletions, the consequences would be relatively minor at DSBs near telomeres, and preferable to chromosome instability resulting from telomere loss. Why human cancer cells are deficient in chromosome healing near telomeres is not known, but could result from excessive degradation of DSBs near telomeres in human cancer cells, since resection in yeast inhibits chromosome healing (Chung et al., 2010; Lydeard et al., 2010).

## THE IMPORTANCE OF TELOMERE LENGTH IN HUMAN DISEASE

The length of telomeres at birth and the rate of telomere shortening in somatic cells can greatly influence the role of loss of telomere function in human disease. The length of telomeres at birth in humans can be affected by mutations in a variety of proteins involved in telomere maintenance, either by affecting telomere-capping function or by directly affecting telomere elongation by telomerase. Definitive evidence that defects in telomere maintenance are associated with human genetic disease come from studies of the genetic disease Dyskeratosis Congenita, which results in early death from bone marrow failure, pulmonary complications, or malignancy (Mason and Bessler, 2011). Dyskeratosis Congenita has now been shown to result from mutations in a number of different telomerase components, including dyskerin, TERC, and TERT, as well as the shelterin component TIN2, which result in telomere shortening and reduced proliferative capacity of cells. Genetic diseases other than Dyskeratosis Congenita have now also been attributed to shortened telomeres, some of which have phenotypes that overlap with Dyskeratosis Congenita, including idiopathic pulmonary fibrosis, Coats plus, aplastic anemia, and liver disease (Savage and Bertuch, 2010; Anderson et al., 2012; Armanios, 2012). Cardiovascular disease has also correlated with telomere length, although the mechanism involved has yet to be clearly established (Panayiotou et al., 2010; Saliques et al., 2010).

In addition to mutations in proteins that affect telomere length, human disease can also be influenced by factors that affect the rate of telomere shortening after birth. An increased rate of telomere shortening can result from either excessive division of adult stem cells, which do not express sufficient telomerase to compensate for telomere loss during cell division. Alternatively, an increased rate of telomere loss can also be caused by factors that increase the amount of telomere loss during cell division, including inflammation and oxidative stress (von Zglinicki, 2002). The effect of life-style on telomere length was first demonstrated by the shortened telomeres in persons under stress (Epel et al., 2004). Subsequent studies have found that a variety of life-style factors can influence telomere length, including smoking, alcohol abuse, and exercise (Lin et al., 2012).

## TELOMERE LOSS AND GENETIC DISEASE

Our observation that DSBs near telomeres in mouse ES cells result in telomere loss and chromosome instability has important implications for human evolution and genetic disease. Subtelomeric regions in humans are highly dynamic, containing large numbers of relatively recent duplications shared by different chromosomes, leading to the proposal that subtelomeric regions serve as birthplaces for new genes (Mefford and Trask, 2002). The subsequent analysis of these duplications led to the conclusion that they result from a high frequency of translocations between subtelomeric regions on different chromosomes (Linardopoulou et al., 2005). This observation is consistent with the demonstration by our lab and others that translocations are a common mechanism of telomere acquisition for chromosomes that have lost a telomere (Artandi et al., 2000; Lo et al., 2002b; Sabatier et al., 2005). The sensitivity of subtelomeric regions to DSBs is also consistent with the fact that many human genetic diseases result from alterations near the ends of chromosomes (Mefford and Trask, 2002), and that these rearrangements are typical of the rearrangements that result from telomere loss. Terminal deletions with telomeric repeat sequences added directly on to the end of the broken chromosome, i.e., chromosome healing, are responsible for a variety of genetic diseases (Fortin et al., 2009; Bonaglia et al., 2011). In addition, terminal deletions in combination with inverted repeats and translocations, classic hallmarks of telomere loss involving one or more B/F/B cycles, are also known to result in numerous human genetic diseases (Hoo et al., 1995; Cotter et al., 2001; South et al., 2006; Zuffardi et al., 2009; Yu and Graf, 2010). Understanding the mechanisms responsible for the sensitivity of telomeric regions to DSBs will therefore provide new insights into mechanisms of human genetic disease, as well as aging (senescence) and cancer (genomic instability).

## Conflict of Interest Statement

The authors declare that the research was conducted in the absence of any commercial or financial relationships that could be construed as a potential conflict of interest.
